# Wandsworth Guardians and Administrative Reform

**Published:** 1913-05-17

**Authors:** 


*To the Editor of* THE HOSPITAL.

London.

### Wandsworth Guardians and Administrative Reform.

*To the Editor of* THE HOSPITAL.

SIR,—Dr. Parsons' objections to parts of the administrative reform scheme suggested to the Wandsworth Guardians in your issue of the 3rd inst. made interesting reading. They pertain to details and can be readily answered. The Wandsworth Union is a large one, but the buildings are not far from each other and facilities for travel abound. Besides, a salary of £900 would enable the medical superintendent to possess a motor-car if he disdains the ordinary vehicles. The actual number of beds does not matter, but only the number of beds occupied by those requiring medical attention. Few beds in the workhouse are utilised for the sick, who are transferred to the infirmaries on the very first complaint of illness. The masters of the workhouse and Tooting Home will remain, only their administration will now be under the supervision of one controlling authority. The scheme urged the proper classification of the cases. The chronic cases in St. John's will not then need a medical superintendent. The seniors at the salaries suggested—only a little lower than that paid to most medical superintendents—will be quite capable and experienced men. Dr. Parsons' gibe at the probability of the "junior staff" doing the surgical work is unjust.

The whole scheme was framed so as to attract the best men for the senior posts. If the medical superintendent performed the major operations, delegating the minor surgery to his capable senior, he would easily be able to attend to his administrative duties. It is not a question of "one superintendent less," but one of efficient administration from the public point of view. The scheme aims at making the indoor Poor-Law service more attractive by asserting the responsibilities of the senior resident medical officers by increasing their pay while securing administrative efficiency. If the Society of Medical Superintendents would insist on their seniors being well paid instead of being sweated at a paltry £175 a year, they would be conferring a real benefit on the service. But years have passed, and this has not been done.

The objections to the scheme are far outweighed by the advantages. The resulting uniformity of administration alone would be a striking improvement. Every administrative factor would be facilitated. Anyone acquainted with the gross waste of public money in "transfers" alone from institution to institution (from one to the other and back again, and so on *ad nauseam*) resulting from a duality of control would welcome a change. If St. James' Infirmary be made the receiving and classifying institution the medical superintendent must have the subsequent control, otherwise patients could be discharged immediately on arrival at the other institution, only perhaps to be readmitted to him again next day. The administrative work would thus be doubled. The Wandsworth Guardians are a fairly progressive body, and the suggested scheme will commend itself to them.

THE WRITER OF THE PAPER.

London, May 9, 1913.

